# A multilevel and multicenter assessment of health care system capacity to manage cardiovascular diseases in Africa: a baseline study of the Ghana Heart Initiative

**DOI:** 10.1186/s12872-023-03430-5

**Published:** 2023-08-24

**Authors:** Alfred Doku, Lawrence Sena Tuglo, Felix Chilunga, Juliette Edzeame, Ron J.G. Peters, Charles Agyemang

**Affiliations:** 1 Department of Medicine and Therapeutics, College of Health Sciences, University of Ghana Medical School, Accra, Ghana; 2 Department of Public & Occupational Health, Amsterdam UMC, Amsterdam Public Health Research Institute, University of Amsterdam, Amsterdam, The Netherlands; 3 Department of Nutrition and Dietetics, School of Allied Health Science, University of Health and Allied Sciences, Ho, Ghana; 4 Department of Epidemiology, School of Public Health, Nantong University, 9 Seyuan Road, Nantong, Jiangsu China; 5 Department of International Services, Deutsche Gesellschaft fur Internationale Zusammenarbeit, Accra, Ghana; 6 Department of Cardiology, Amsterdam UMC, University of Amsterdam, Amsterdam, The Netherlands

**Keywords:** Multilevel and multicenter assessment, Health care system capacity, Cardiovascular diseases, Ghana Heart Initiative

## Abstract

**Introduction:**

Cardiovascular diseases (CVD) remain the leading cause of death worldwide, with over 70% of these deaths occurring in low- and middle-income regions such as Africa. However, most countries in Africa do not have the capacity to manage CVD. The Ghana Heart Initiative has been an ongoing national program since 2018, aimed at improving CVD care and thus reducing the death rates of these diseases in Ghana. This study therefore aimed at assessing the impact of this initiative by identifying, at baseline, the gaps in the management of CVDs within the health system to develop robust measures to bolster CVD management and care in Ghana.

**Methods:**

This study employed a cross-sectional study design and was conducted from November 2019 to March 2020 in 44 health facilities in the Greater Accra region. The assessment covered CVD management, equipment availability, knowledge of health workers in CVD and others including the CVD management support system, availability of CVD management guidelines and CVD/NCD indicators in the District Health Information Management System (DHIMS2).

**Results:**

The baseline data showed a total of 85,612 outpatient attendants over the period in the study facilities, 70% were women and 364(0.4%) were newly diagnosed with hypertension. A total of 83% of the newly diagnosed hypertensives were put on treatment, 56.3% (171) continued treatment during the study period and less than 10% (5%) had their blood pressure controlled at the end of the study (in March 2020). Other gaps identified included suboptimal health worker knowledge in CVD management (mean score of 69.0 ± 13.0, p < 0.05), lack of equipment for prompt CVD emergency diagnosis, poor management and monitoring of CVD care across all levels of health care, lack of standardized protocol on CVD management, and limited number of indicators on CVD in the National Database (i.e., DHIMS2) for CVD monitoring.

**Conclusion:**

This study shows that there are gaps in CVD care and therefore, there is a need to address such gaps to improve the capacity of the health system to effectively manage CVDs in Ghana.

## Introduction

More people die from cardiovascular diseases (CVDs) worldwide than from any other cause – an estimated 17.9 million people in 2019, accounting for 32% of all global deaths [1]. Of these deaths, 85% were due to heart attacks and stroke [1]. CVDs disproportionately affect low- and middle-income countries (LMICs), and in many countries, the economic and social burden is highest amongst the poor and disadvantaged groups [2]. In 2019, more than three-quarters of the 17.9 million CVD deaths and 87% of CVD-related disabilities worldwide occurred in LMICs [3]. The deteriorating pattern has continued in an upwards trend in LMICs in the past two decades [4]. Sub-Saharan Africa (SSA) remained the only part of the world where CVD deaths increased between 1990 and 2013 whereas, CVD mortality rates were noted to decline or at worst, be steady in other regions of the world [3, 5]. The burden of CVDs is projected to double in SSA by 2030 [6].

In Ghana, the rapid epidemiological transition has been unprecedented with a shift of increasing morbidity and mortality from communicable diseases to both communicable and non-communicable diseases (known as the double burden of disease) [5, 7]. CVDs have been the leading cause of death from all causes since the year 2016 with strokes and hypertensive heart diseases being the foremost on the list according to the District Health Information Management System of Ghana over the past five years [8, 9]. Effective methods for reducing the burden of CVD have been well documented in the literature and include population-wide interventions to reduce overall risk factor exposure, individual approaches to modify risk factors for high-risk individuals (including people with diabetes) and prompt diagnosis and management of CVD events [10].

Concerted efforts by international organizations such as World Health Organization in collaboration with national bodies have sought to implement these strategies at the population and individual level in various regions of the world with some success [4]. For instance, in some high-income countries, efforts have been successful in tackling commercial determinants of behavioural risk factors for CVDs (such as alcohol and tobacco), strengthening health system capacity in CVD management and have hence seen a decline in CVD morbidity and mortality rates [11]. Despite existing structures in the health care system to ameliorate the burden of CVD in Ghana, studies have highlighted the increasing devastation caused by CVD in Ghana [12]. This observation, therefore, emphasizes the need to assess the health system for gaps in CVD management and propose practical and sustainable remedies to address the chasm.

This study, as part of the Ghana Heart Initiative (GHI) project, thus sought to evaluate the health system in Ghana in this capacity for CVD management. This involved assessing the health system’s capacity and readiness for CVD care to strengthen and or revise existing efforts in place, and to put in new measures to bolster efforts for CVD management in Ghana.

## Methodology

This study employed a cross-sectional study design; conducted between November 2019 to March 2020 across 44 health facilities in the Greater Accra region. These facilities were purposively selected from the 44-implementing public/government and quasi-government facilities of the GHI in the Greater Accra region.

### Data collection

The research team utilized a combination of desk review, stakeholder consultations and facility- assessment of equipment and human resources capacity in CVD management. The data collection involved establishing CVD burden in participating health facilities by recording data of patients who were newly diagnosed with hypertension, stroke, myocardial infarction and heart failure at the outpatients’ departments (OPDs), consulting rooms and medical wards. New cases of CVDs and risk factors were recorded by doctors and consulting room nurses and subsequently validated by health information officers (HIOs) in the various health facilities. This procedure provided an efficient way of engaging healthcare employees e.g., doctors and nurses, and HIOs on the project objectives and indicators.

The adopted method was effective in both facilities that use paper-based folders and those that used electronic medical records. A baseline orientation was done to train frontline staff mainly doctors, OPD/ER nurses and health information officers to enable them to collect CVD data for the study. The study conducted a knowledge-based assessment of health workers of different cadres across different levels of care on CVD management. Questions ranged from basic diagnosis and management of CVD emergencies as well as questions on basic life support. The set of questions and degree of difficulty was different for the three cadres of staff because of differences in basic qualifications, job description and expected knowledge base. Nurses, physician assistants and doctors answered 30, 40 and 40 multichoice questions for 45 min respectively.

Facilities were stratified according to their level of care i.e., CHPS Compounds and health centres (this is the first level of care), polyclinics (the second level and referral facilities for health centres), sub-district and district hospitals. However, data collection at the polyclinics was further stratified as those that admit and those that do not admit cases. The third level of stratification was hospitals that see primary and secondary-level cases. These facilities serve as the referral points for the polyclinics and hospitals. The last category is tertiary facilities, which include regional hospitals, teaching hospitals and higher-level quasi-government hospitals (e.g., the Trust Hospital, the Police Hospital and 37 Military Hospitals.

### Questionnaire

Due to the different sizes and roles of each facility, four different questionnaires were developed and validated for use in data collection at the various facilities. These various facilities are CHPS compounds (category 1); health centres (category 2); polyclinics, District Hospitals, Municipal Hospitals, General Hospitals (category 3) and Regional Hospitals, high-level quasi-government, and tertiary facilities (category 4).

### Data analysis

Twenty-seven (27) facilities completed the baseline CVD data collection within the recommended timelines, 44 facilities provided data on functional equipment availability and 36 facilities provided staff for CVD knowledge assessment and training. The data obtained were collated and cleaned with Microsoft Excel 2016 and imported to SPSS version 25 for analysis. Descriptive analysis was performed to summarize variables by frequencies and percentages. We performed an association between blood pressure control against baseline characteristics of responders using Fisher’s exact test or the Chi-squared test where appropriate, and those with significant variables (p < 0.05) were included in logistic regression analysis.

### Ethical clearance

Ethical approval was obtained (GHS-ERC: 018/05/19) from Ghana Health Service on 19th July 2019 to assess the capacity of the health system to manage CVD. The research methods were carried out following relevant Ghana Health Service guidelines and regulations. Informed consent was obtained from all participants before data collection.

## Results

### Baseline characteristics of OPD attendance

A total of 85,612 baseline OPD attendance data were retrieved among patients aged 18 years and above. They were more females 70.0% compared to males. Most 44.0% aged between 18 and 34 years. Based on the facility type, the majority (n = 72,544; 84.7%) were from polyclinics/ district/ municipal/ general hospitals (Table 1).

**Table 1:** Baseline characteristics of OPD attendance

Variable	Frequency	Percentage
Gender		
Female	59,668	70.0
Male	25,944	30.0
Age group (years)		
18–34	37,834	44.0
35–59	30,738	36.0
> 59	17,040	20.0
Facility type		
CHPS compounds	393	0.50
Health Centers and some Polyclinics	12,675	14.8
Polyclinic/ District/ Municipal/ General Hospitals	72,544	84.7

Data are presented as frequencies and percentages

### Prevalence of newly diagnosed hypertension

Out of a total of 85,612 baseline data, 364 (0.43%) patients were newly diagnosed with hypertension. The prevalence was higher among females, those aged above 59 years and accessed healthcare in primary facilities, 0.45%, 1.05% and 0.50% respectively (Table 2).

**Table 2:** Prevalence of newly diagnosed hypertension

Variable	Frequency	Prevalence
Gender		
Female	268	0.45
Male	96	0.37
Age group (years)		
19–34	49	0.13
35–59	136	0.44
> 59	179	1.05
Facility type		
Primary	65	0.50
Secondary	299	0.41

Data are presented as frequencies and prevalence

### Baseline characteristics of respondents against blood pressure control

Out of the newly diagnosed hypertensives, there were more females (73.6%) than males, and the difference was statistically significant (*p* = 0.019). The total average age was 55.55 ± 17.43 years. According to the classification of SBP and DBP, the majority (n = 181; 49.7%) and (n = 245; 67.3%) were in grade 2 and grade 3 for SBP and DBP respectively. No significant differences were found between the blood pressure control for age, and facility type (*p* > 0.05) (Table 3).

**Table 3:** Baseline characteristics of respondents against blood pressure control

Variable		Blood pressure control		
	Total (n = 364)	No (n = 356)	Yes (n = 8)	p-value
Gender				0.019
Female	268 (73.6)	265 (74.4)	3 (37.5)	
Male	96 (26.4)	91 (25.6)	5 (62.5)	
Age (years)	55.55 ± 17.43	55.64 ± 17.39	51.50 ± 20.17	0.507
Age group (years)				0.596
19–34	49 (13.5)	47 (13.2)	2 (25.0)	
35–59	136 (37.3)	133 (37.4)	3 (37.5)	
> 59	179 (49.2)	176 (49.4)	3 (37.5)	
Facility type				0.142
Primary	65 (17.9)	62 (17.4)	3 (37.5)	
Secondary	299 (82.1)	294 (82.6)	5 (62.5)	
SBP (mmHg)	168.27 ± 18.13	167.99 ± 17.97	180.88 ± 21.95	0.047
SBP classification				0.007
Grade 1 (140–159)	111 (30.5)	109 (30.6)	2 (25.0)	
Grade 2 (160–179)	181 (49.7)	180 (50.6)	1 (12.5)	
Grade 3 (≥ 180)	72 (19.8)	67 (18.8)	5 (62.5)	
DBP (mmHg)	111.83 ± 11.13	111.96 ± 11.32	105.75 ± 5.39	0.123
DBP classification				0.030
Grade 1 (90–99)	28 (7.69)	27 (7.58)	1 (12.5)	
Grade 2 (100–109)	91 (25.0)	86 (24.2)	5 (62.5)	
Grade 3 (≥ 110)	245 (67.3)	243 (68.3)	2 (25.0)	

Data are presented as frequencies with percentages in parentheses and means ± standard deviation. Where appropriate, categorical data were compared using Fisher’s exact test or the Chi-squared test, and continuous data were compared using an independent sample t-test. SBP; Systolic Blood Pressure, DBP; Diastolic Blood Pressure, Grade 1: Mild Hypertension, Grade 2: Moderate Hypertension, Grade 3: Severe Hypertension, Significant at p < 0.05

### Factors associated with blood pressure control

The female gender was 0.21 times less likely to have blood pressure control compared to the male [UOR = 0.21 (95% Cl: 0.05–0.88)]. Those with grade 2 systolic hypertension were 11 times more likely to have their blood pressure control compared to those classified as grade 3 [Adjusted Odds Ratio (AOR) = 11.08 (95% Cl: 1.17–105.30)]. In contrast, those with grade 2 diastolic hypertension had a lower tendency to have their blood pressure control than those in grade 3 [UOR = 0.21 (95% Cl: 0.04–1.19)] (Table 4).

**Table 4:** Factors associated with blood pressure control

Variable	UOR (95% CI)	p-value	AOR (95% CI)	p-value
Gender				
Female	0.21 (0.05–0.88)	0.033	0.28 (0.06–1.31)	0.107
Male	1		1	
SBP classification				
Grade 1 (140–159)	4.07 (0.77–21.56)	0.099	3.18 (0.57–17.84)	0.189
Grade 2 (160–179)	13.43 (1.54-117.09)	0.019	11.08 (1.17–105.30)	0.036
Grade 3 (≥ 180)	1		1	
DBP classification				
Grade 1 (90–99)	0.22 (0.02–2.53)	0.226	0.17 (0.01–2.01)	0.157
Grade 2 (100–109)	0.14 (0.03–0.74)	0.021	0.21 (0.04–1.19)	0.078
Grade 3 (≥ 110)	1		1	

Grade 1: Mild Hypertension, Grade 2: Moderate Hypertension, Grade 3: Severe Hypertension, UOR; Unadjusted Odds Ratio, AOR; Adjusted Odds Ratio, 1; Reference, Significant at p < 0.05

### Assessment of hypertension management

Out of 364 newly diagnosed hypertensive, 302 were put on treatment (antihypertensive drugs) representing 83% and the remaining 62 were managed by lifestyle modifications. Among the 302 patients put on treatment, 171 (56.6%) of them were continuing with the treatment. Eight (5%) of the patients continuing treatment had their BP controlled.

### CVD diagnosis

There were only six (6) recorded cases of CVD of which 4(67%) were stroke cases and 2(33%) were heart failure cases. One of the heart failure patients died whilst on admission.

### Equipment needs assessments

**Fig. 1:**
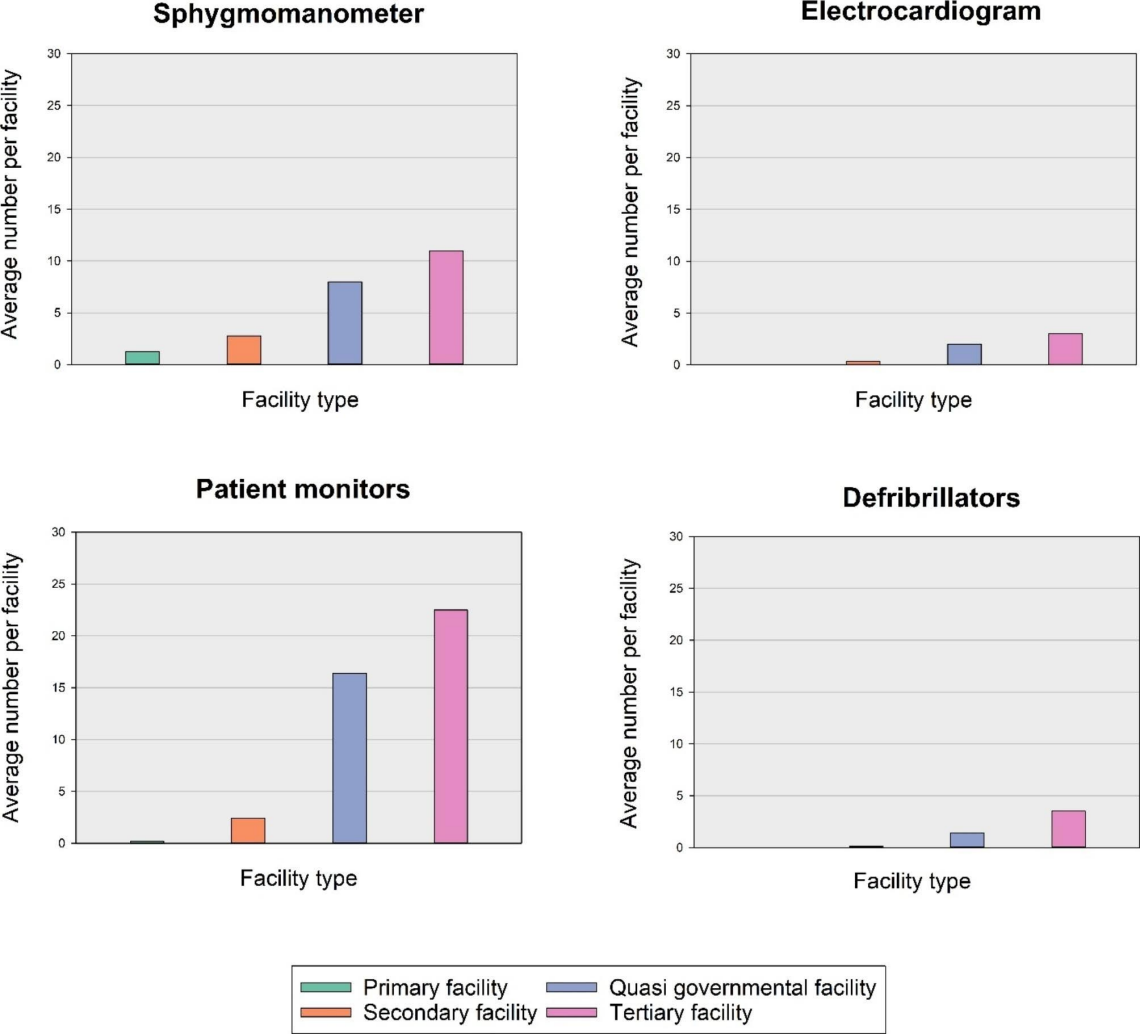
Equipment Need Assessment

Across the primary level of health facilities assessed (such as CHPS compounds and health centres), on average only one functional sphygmomanometer was available to serve clients, with no electrocardiograms or defibrillators present. The secondary level health facilities (district, municipal hospitals and polyclinics) had an average of 3 sphygmomanometers per facility; 1 in 3 facilities had an electrocardiogram machine and 1 in 6 had a defibrillator present at the time of needs assessment. Although the majority of CVD-related health services in Ghana are concentrated in secondary and tertiary centres, a general lack of emergency diagnostic equipment was noted with only an average of 3 sphygmomanometers per secondary facility, 11 per tertiary facility; 1 in 3 secondary health facilities had electrocardiogram vis a vis 3 in tertiary facilities; and 1 in 6 secondary health facilities had a defibrillator as compared to 3.5 per tertiary facility at the time of needs assessment. The situation was even direr in the primary level health care of participating institutions where only one sphygmomanometer and no ECGs or defibrillators were noted present (Fig. 1).

### Assessment of the health workers’ knowledge of risk factors and CVDs

Out of a total of 260 participants who completed the questionnaire, women were 191(73%). Of the total number who took part in all three questionnaires, 64 were medical doctors, 52 were Physician Assistants and the remaining 144 (55%) were nurses; from 36 health facilities in 13 districts of the Greater Accra Region. Forty (15%) of the trainees were working in primary level facilities (CHPS Compound and Health Centers), 157 (60%) from district hospitals and policlinics and the rest (25%) from teaching hospitals and other tertiary facilities. Out of 260 conveniently sampled health workers, which include doctors, nurses and physician assistants, an average score of 68.4 (SD ± 14.9) was noted. There was a significant difference in scoring among the different cadres of work with specialists and medical officers having better scores than the other cadres (p < 0.05). The skill set of health professionals was however not objectively assessed.

### Other health system gaps in CVD management identified during the assessments

The assessment showed that aside from the Standard Treatment Guidelines, there were no national CVD guidelines or treatment protocols. There were no national or regional CVD support systems or call centres available to provide topside support to lower-level facilities to improve care. The health system also lacked dedicated CVD quality improvement interventions or strategies such as supportive supervision and mentorship programs to improve CVD management. The DHIMS2 had aggregated data on hypertension and CVDs, which was not enough to inform decisions and policies. For hypertension, the data did not disaggregate information into total burden/prevalence, proportion of those newly diagnosed, proportion on treatment and proportion with controlled hypertension. Hypertension-mediated target organ damage (e.g., retinopathy, nephropathy, hypertensive heart diseases, etc.) data were not available. In addition, heart failure and stroke, the leading causes of death in Ghana from the DHIMS2, did not have disaggregated information or data on the type of stroke (hemorrhagic or infarction) and the cause of heart failure (hypertensive, ischemic, valvular, congenital, cardiomyopathies, etc.).

## Discussion

Health system strengthening is the most appropriate response to addressing the health and health system challenges of any country. This analysis assessed the health system capacity and readiness for CVD care to strengthen and/or revise and boost existing efforts in place for CVD management in Ghana. The study found that equipment, health workers’ knowledge of CVD risk factors and health system gaps in CVD management situations in Ghana is dire and requires urgent attention if outcomes of CVD are to be improved. Efforts to bolster CVD care in Ghana however hinges on first, identifying relevant gaps in CVD management in the health care system and implementing practical, sustainable measures to close those gaps and improve health outcomes for patients with CVD in Ghana. This was the goal of the GHI baseline needs assessment study. The needs assessment in CVD care and management highlighted major gaps in health care delivery in Ghana.

First, there was sub-optimal health capacity in CVD management across health facilities in the Greater Accra region. A baseline assessment was done among different cadres of healthcare workers in various health facilities on knowledge of risk factors and CVD showed only a fair level of knowledge of CVD emergency management. As expected, specialists scored the highest, followed by medical officers/residents and then physician assistants and nurses. Specialist in Ghana is a top-ranked medical position as compared with the other positions like the medical officers/residents and the PAs and nurses. This could explain their highest knowledge score in CVD management. Similar findings have been established elsewhere. A study conducted in Iran among female health workers showed a score of 56% [13], and less than 50% of nurses in a provincial hospital in China knew CVD risk factor targets [14].

A similar study conducted in Ukraine among family physicians showed a very low level of knowledge of CVD guidelines with 85.8% scoring below the acceptable knowledge level [15]. These studies demonstrate a lower level of knowledge among health professionals in CVDs albeit higher average scores in our study.

Additionally, the health care system in Ghana has different levels of care with no convergent, standardized national guidelines in CVD management. The Pan-African Society of Cardiology has intimated that only 25.8% of countries in Africa have national hypertension guidelines [11], with an even smaller percentage for CVD. A standardized guideline on CVDs will provide health workers at all tiers of health care with a basis for mutual understanding, indicating common protocols to measure and evaluate performance as well as improve patient CVD outcomes. It will serve as a guide to practice, providing boundaries of care and set requirements for referral to higher facilities when necessary.

Although standard treatment guidelines instituted by the Ministry of Health (MoH) are present in some health facilities in Ghana, the practice of CVD management leaves room for improvement as divergent protocols are employed by different health workers. A standardized, national CVD guideline, backed by training of health professionals to build capacity, is thus paramount in achieving comprehensive care at different levels of health care in Ghana and is proposed to significantly improve CVD outcomes in Ghana. Healthcare facilities across all tiers of care grossly lacked the appropriate medical equipment and supplies to successfully manage CVD emergencies promptly. Medical equipment is crucial in the detection, management and prevention of illness and diseases. They are used to promote health services. The lack of appropriate medical equipment is supported by an assertion made by Bosu et al., [16] that the country is lacking medical equipment, especially at district level and teaching hospitals. The absence of this basic diagnostic and therapeutic equipment in CVD care at all levels of health care threatens the effective management of CVD emergencies promptly with prospects of increased mortality and or poor health outcomes.

The lack of basic diagnostic and management equipment for risk factors and CVD has been identified as one of the key gaps for CVD management in Africa and a key recommendation by the authors for achieving hypertension control and overall CVD management in Africa [11]. Strengthening the capacity and resources of the primary health care centres to be able to contribute to assessing CVD risk factors and initiating basic prevention and treatment interventions is fundamental to CVD care in Ghana as they are often the first point of call for some sections of the population, especially in the rural and underserved areas.

There was 100% opportunistic screening for hypertension within the health facilities assessed. The prevalence of new hypertension cases is higher among lower-tier facilities. This is partly because they are the first point of call when the larger population want to visit facilities for the first time, and because the lower-tier facilities probably showed higher commitment in recording cases for the project. In addition, blood pressure control was very low (5%). Blood pressure control is very low across Africa (< 10%) [17]. There are several barriers and factors to account for this low level of control. Gender, educational level, and co-morbidities have been identified as factors associated with low blood pressure control in the Greater Accra Region of Ghana [12]. However, our study found mainly gender and blood pressure levels (diastolic and systolic levels) before the initiative on therapy. The authors recommend further robust studies to unravel the barriers to achieving better blood pressure control in Ghana.

As CVD cases keep rising in Ghana, there is unfortunately inadequate support for primary healthcare facilities to track and manage these effectively as identified by the findings of the needs assessment [12]. Even though many gains have been made in maternal and child health by using the CHPS concept to bridge access and improve care outcomes, the same is yet to be said of CVDs. The authors are recommending the need to broaden the scope for primary healthcare centres such as CHPS and health centres to form the nucleus for early detection of CVD risk factors and awareness creation as a means of reducing late CVD complications and poor outcomes that are often seen in most health facilities, especially at the secondary and tertiary levels. One of the key interventions introduced by the GHI was the introduction and establishment of a CVD call support centre in a tertiary/quaternary hospital manned by specialists. The objective of this is to provide support to healthcare providers at the primary level to enable them to effectively manage and improve outcomes of patients with CVDs and also to facilitate prompt referrals.

### Implication for public health practice

Needs assessment of the healthcare system for CVD management is key to improving outcomes. This assessment made it possible in identifying the gaps that exist in the healthcare system in Ghana. One key gap is the lack of medical equipment across all levels of care. The GHI intervention after the needs assessment supplied several pieces of equipment including BP monitors, ECG machines, defibrillators, etc. across all levels of care in the 44 implementing health facilities in the Greater Accra region. The CVD support centre is an important intervention that further strengthens and streamlines the referral system and further bolsters the confidence and competence of providers at the primary health care levels in improving the quality of CVD care. This was an obvious gap in the Ghanaian healthcare system worth closing. The District Health Information Management Systems (DHIMS) is the main data collection and health reporting tool used by Ghana Health Service in tracking CVDs and other health indicators in Ghana.

The inadequate CVD-related indicators in the DHIMS 2 were also identified as a major gap that needed to be addressed. For instance, there were only 11 indicators that were reported on from the various health facilities into DHIMS and these indicators made it difficult and sometimes even impossible to evaluate the quality of care provided and put in place effective interventions to address them. This has also been identified as one of the key gaps in CVD care in Africa [17]. The GHI intervention has improved the data CVD data quality by introducing and standardizing CVD-related data to facilitate effective collection via the DHIMS 2 platform and utilization for improved care outcomes across the country.

### Study limitations

The actual prevalence rate of newly diagnosed hypertension patients may be higher in the population than our current finding since the total attendance (the base/denominator) reported included only newly diagnosed hypertensives. Our study did not assess other CVD risk factors such as diabetes, hyperlipidemia, smoking status, etc. The follow-up period for newly diagnosed hypertension was up to only three months.

### Conclusion and recommendation

This study sought to evaluate the health system in Ghana in the capacity for CVD management by assessing the health system’s capacity and readiness for CVD care to strengthen and or revise existing efforts in place, and to put in new measures to bolster efforts for CVD management has identified several gaps in CVD management in Ghana. Based on the findings, we recommended the following effective measures and strategies to the appropriate agencies in Ghana for improving CVD care:

1. Develop standardized national guidelines on CVD management for different levels of health care in Ghana and put mechanisms in place to ensure its adherence.
2. Put mechanisms in place to ensure that healthcare providers are regularly trained and retrained to build their capacity in the management of CVD.
3. Ensure that all levels of health facilities are supplied with appropriate equipment to enable early diagnosis and management of CVD emergencies.
4. Establish a CVD support centre and facilitate its use as one of the approaches to improving the referral system and supporting primary health care providers to effectively manage and improve the outcomes of CVD patients in Ghana.
5. Integrate quality improvement strategies such as supportive supervision and mentorship programs into various health interventions/programs to improve CVD outcomes.
6. Improve the list of CVD-related indicators in the DHIMS-2 and data collection process on CVD to ensure accurate and reliable data for decision-making and policy development on CVDs in Ghana.

## Data Availability

Data is available from the corresponding author upon reasonable request.

## Abbreviations

AOR: Adjusted Odds Ratio
CHPS: Community-Based Health Planning and Services
Cl: Confidence Interval
CVD: Cardiovascular Diseases
DBP: Diastolic Blood Pressure
DHIMS2: District Health Information Management System
ECG: Electrocardiogram
GHI: Ghana Heart Initiative
GHS-ERC: Ghana Health Service Ethics Review Committee
HIOs: Health Information Officers
LMICs: Low- and middle-income countries
MOH: Ministry of Health
NCD: Non-Communicable Disease
OPDs: Outpatients’ Departments
SBP: Systolic Blood Pressure
SSA: Sub-Saharan Africa
UOR: Unadjusted Odds Ratio
